# Low Pulmonary Function in Individuals with Impaired Fasting Glucose: The 2007-2009 Korea National Health and Nutrition Examination Survey

**DOI:** 10.1371/journal.pone.0076244

**Published:** 2013-09-27

**Authors:** Yun Jeong Lee, Na Kyung Kim, Ju Yean Yang, Jung Hyun Noh, Sung-Soon Lee, Kyung Soo Ko, Byoung Doo Rhee, Dong-Jun Kim

**Affiliations:** 1 Department of Internal Medicine, Inje University College of Medicine, Busan, Korea; 2 Department of Internal Medicine, Samsung Medical Center, Sungkyunkwan University School of Medicine, Seoul, Korea; German Diabetes Center, Leibniz Center for Diabetes Research at Heinrich Heine University Duesseldorf, Germany

## Abstract

**Objective:**

To investigate the association between fasting plasma glucose level and pulmonary function.

**Research Design and Methods:**

Nutritional information, pulmonary function data, and laboratory test data from 9,223 subjects from the fourth Korea National Health and Nutrition Examination Survey were examined. The participants were divided into five groups according to fasting plasma glucose (FPG) level: normal fasting glucose (NFG)1, FPG <90 mg/dl; NFG2, FPG 90-99 mg/dl; impaired fasting glucose (IFG)1: FPG 100-109 mg/dl; IFG2, FPG 110-125 mg/dl; and diabetes, FPG ≥126 mg/dl and/or current anti-diabetes medications.

**Results:**

After adjustment for several variables, the percentage of predicted forced vital capacity(FVC%) decreased with increasing fasting plasma glucose level in both sexes[men: (mean ± SEM) 92.0±0.3 in NFG1; 91.9±0.3 in NFG2; 92.0±0.4 in IFG1; 90.2±0.7 in IFG2; and 89.9±0.5 in diabetes, P = 0.004; women: 93.7±0.3 in NFG1; 93.7±0.3 in NFG2; 93.1±0.5 in IFG1; 91.1±0.9 in IFG2; and 90.7±0.6 in diabetes, P<0.001]. A logistic regression analysis found that IFG2 and diabetes were independently associated with the lowest quintile of predicted FVC% (IFG2: odds ratio [95%CI], 1.50 [1.18-1.89], P = 0.001; diabetes: 1.56 [1.30-1.88], P<0.001) using NFG1 as a control.

**Conclusions:**

The current data suggest that forced vital capacity may begin to decrease in the higher range of IFG.

## Introduction

Diabetes mellitus is known to affect many aspects of the human body including the retina, nerves, kidneys, and the cardiovascular system as a result of micro- and macro-angiopathy [1]. The alveolar-capillary network in the lungs is a large microvascular unit that may be affected by microangiopathy similar to other organ systems [2]. Many cross-sectional studies have found that diabetes is associated with low forced vital capacity (FVC) and low forced expiratory volume (FEV); reductions in FVC are more consistent than reductions in FEV [3-7]. Moreover, several longitudinal studies have found that pulmonary dysfunction is a possible complication of diabetes as well as a possible predictor of the development of diabetes [8,9].

Many studies have shown that pre-diabetes (impaired fasting glucose [IFG] or impaired glucose tolerance [IGT]) may be associated with the development of diabetic complications [10,11]. However, almost all studies of the association between pulmonary function and glucose tolerance status compared diabetes and non-diabetes; very few have investigated this association in a non-diabetic range [5,6,12].

The Korean Center for Disease Control and Prevention (CDC) has conducted a series of Korea National Health and Nutrition Examination Surveys (KNHANES) since 1998. Pulmonary function tests were included in the fourth KNHANES (2007–2009). Using the nationwide representative KNHANES sample, we investigated the association of pulmonary function with fasting plasma glucose level in a non-diabetic range and tried to estimate the cut-off value of fasting plasma glucose (FPG) associated with the beginning of a decrease in pulmonary function.

## Research Design and Methods

### Ethics statement

This study was approved by the institutional review board of Ilsan Paik Hospital, South Korea (IB‑1210-032). After approval of the study proposal, the KNHANES dataset was made available at the request of the investigator. Because the dataset did not include any personal information and participants’ consent had already been given in the KNHANES, our study was exempted from participant consent by the board.

### Study population and data collection

KNHANES is a cross-sectional and nationally representative survey of the health and nutritional status of the Korean population. The Korean CDC has conducted a series of KNHANES. The health examinations in the fourth KNHANES consisted of a physical examination, a questionnaire about medical history and health-related lifestyle, anthropometric measurements, and biochemical laboratory measurements. Health-related questions included smoking, alcohol drinking, and current medication. Participants with a disease responded to additional questions concerning the duration of disease and specifics of the medications used. The questionnaire also included history of chronic obstructive pulmonary disease, bronchial asthma, coronary heart disease, cerebrovascular disease, and cancer, diagnosed by a doctor. For biochemical measurements, participants underwent blood sampling after 8 h of overnight fasting. In addition, pulmonary function tests were included. This study evaluated the laboratory tests, health information, and pulmonary function test results from this data set.

A stratified multistage clustered probability sampling design was used to select a representative sample of civilian, non-institutionalized Koreans. Six hundred sampling frames (12,000 households) from primary sampling units were randomly sampled, and 31,705 individuals from these sampling frames were included in the fourth KNHANES. Of these survey candidates, 23,632 persons participated in the study (participation rate, 74.5%). After exclusion of persons aged ≤18 years old, 12,631 persons had available laboratory and pulmonary function tests. We excluded

3,408 persons with unacceptable spirometry performances and only analyzed data from 9,223 individuals with two or more acceptable spirometry performances.

### Health examination survey and laboratory tests

Height and weight were measured using standardized techniques and equipment. Height was measured to the nearest 0.1 cm using a portable standiometer (Seriter; Bismarck, ND) and weight was measured to the nearest 0.1 kg using a Giant-150N calibrated balance-beam scale (Hana; Seoul, Korea). Body mass index (BMI) was calculated by dividing weight in kilograms by height in meters squared (kg/m^2^). Systolic blood pressure (SBP) and diastolic blood pressure (DBP) were measured by standard methods using a sphygmomanometer with the patient in a sitting position. Three measurements of all subjects were taken at 5-min intervals and the average of the second and third measurements was used in the analysis. Blood samples were collected in the morning after fasting for at least 8 h. FPG was measured in a central and certified laboratory using an Advia 1650 (Siemens, USA). HbA1c was measured using a high-performance liquid chromatography (HPLC) assay with a Bio-Rad Varian II (Bio-Rad, Hercules, CA, USA). The methodology was aligned with the Diabetes Control and Complications Trial (DCCT) and National Glycohemoglobin Standardization Program (NGSP) standards [13].

### Spirometry

A Model 1022 Spirometer (SensorMedics; USA) was used for pulmonary function testing. Measurements of FEV1 and FVC were performed in accordance with the recommendations of the American Thoracic Society [14]. The predicted FEV1 and FVC for each subject were calculated using published prediction equations. For men, predicted FVC = -4.8434 - 0.00008633 x Age (years)^2^ + 0.05292 x Height (cm) + 0.01095 x Body weight (kg) and predicted FEV1 = -3.4132 - 0.0002484 x Age (years)^2^ + 0.04578 x Height (cm) ; for women, predicted FVC = -3.0006 - 0.0001273 x Age (years)^2^ + 0.03951 x Height (cm) + 0.006892 x Body weight (kg) and predicted FEV1 = -2.4114 - 0.0001920 x Age (years)^2^ + 0.03558 x Height (cm) [15]. Predicted FVC% and predicted FEV1% were calculated by dividing the FVC and FEV1 by the predicted FVC and FEV1, respectively.

### Five FPG groups

The participants in this study were divided into five groups according to FPG; normal fasting glucose (NFG)1: FPG <90 mg/dl; NFG2: FPG 90-99 mg/dl; impaired fasting glucose (IFG)1: FPG 100-109 mg/dl; IFG2: FPG 110-125 mg/dl; and diabetes: FPG ≥126 mg/dl or treatment with anti-diabetes drugs or a history of diabetes diagnosed by a physician. Because Korean studies have found that IFG2 patients show clinical characteristics different to those of IFG1 patients [16], the 2011 clinical practice guidelines for type 2 diabetes in Korea recommend different screening methods according to IFG stage (stage 1: FPG 100-109 mg/dl; stage 2: FPG 110–125 mg/dl) [17]. Thus, for the current study, participants were divided into two IFG groups according to the above criteria.

### Statistical analyses

**Figure 1:**
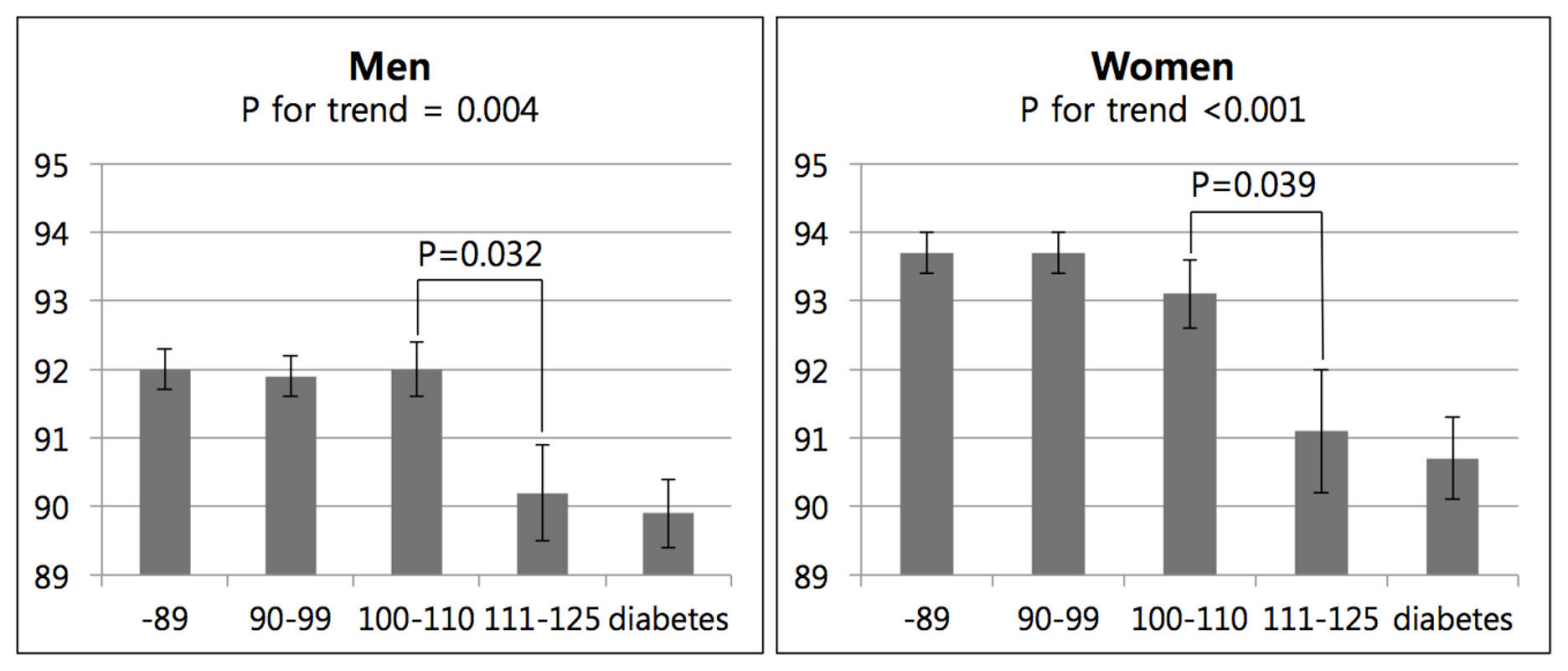
Predicted FVC% by fasting plasma glucose. Adjusted for age, smoking, exercise, and waist circumference.

Data are presented as means or % ± SEM. Statistical analyses were performed using the SPSS software (ver. 18.0 for Windows; SPSS, Chicago, IL, USA). Clinical characteristics were compared according to sex using the independent t test and χ^2^ test (Table 1). Predicted FEV1% and predicted FVC% were compared by ANCOVA according to FPG level, before and after adjustment for age, exercise, smoking, and waist circumference(Figure 1; Table 2). To determine the parameter(s) associated with the lowest quintile of predicted FVC% (<83%) or the lowest quintile of predicted FEV1% (<82%) a logistic regression analysis was conducted with age, sex, exercise, smoking, fasting plasma glucose level, and waist circumference as variables (Table 3). Two-tailed analyses were conducted and a value of P<0.05 was deemed to indicate statistical significance.

**Table 1:** Clinical characteristics of study population aged 19 years and older.

	Men (n=4119)	Women (n=5104)	Total (n=9223)
Age (years)	49.2 ± 0.2	50.7 ± 0.2	50.0 ± 0.2
Past smoking/current smoking (%)	39.3/43.9	5.3/6.5	20.5/23.2
Heavy alcohol drinking (%)	15.2	2.4	8.1
Regular exercise (%)	19.4	14.6	16.7
Body mass index (kg/m^2^)	24.4 ± 0.1	24.0 ± 0.1	24.2 ± 0.1
Waist circumference (cm)	85.9 ± 0.1	81.2 ± 0.1	83.3 ± 0.1
Systolic blood pressure (mmHg)	121.3 ± 0.2	117.2 ± 0.3	119.1 ± 0.2
Diastolic blood pressure (mmHg)	80.0 ± 0.2	75.2 ± 0.1	77.3 ± 0.1
Fasting plasma glucose (mg/dl)	100.3 ± 0.4	97.1 ± 0.3	98.5 ± 0.2
Impaired fasting glucose/diabetes (%)	24.4/11.3	17.5/9.1	20.6/10.1
Predicted FVC%	91.6 ± 0.2	93.3 ± 0.2	92.5 ± 0.1
Predicted FEV1%	90.0 ± 0.2	93.4 ± 0.2	91.9 ± 0.1

Data, mean or % ± SEM. Heavy alcohol drinking, ≥ x4 alcohol drinking/week. Regular exercise, ≥ x5 exercise/week. Impaired fasting glucose, 100 ≤ fasting plasma glucose ≤ 125 mg/dl. Diabetes, fasting plasma glucose ≥ 126 mg/dl or current anti-diabetes medication or previous diagnosis of diabetes by doctor. All variables were significantly different between each sex (P<0.001).

**Table 2:** Predicted FEV1 % and predicted FVC % by fasting plasma glucose.

			NFG		IFG		diabetes	P
			-89	90-99	100-109	110-125		
FVC %	No adjustment	Men	93.7±0.4	92.2±0.3	91.4±0.4	88.2±0.7	87.0±0.6	<0.001
		Women	94.4±0.3	93.8±0.3	92.5±0.5	89.9±0.9	89.1±0.6	<0.001
	After adjustment	Men	92.0±0.3	91.9±0.3	92.0±0.4	90.2±0.7	89.9±0.5	0.004
		Women	93.7±0.3	93.7±0.3	93.1±0.5	91.1±0.9	90.7±0.6	<0.001
FEV1 %	No adjustment	Men	91.2±0.4	90.5±0.4	90.0±0.5	87.3±0.8	87.0±0.6	<0.001
		Women	93.5±0.3	93.8±0.3	93.6±0.5	91.9±1.0	92.1±0.6	0.062
	After adjustment	Men	90.1±0.4	90.3±0.3	90.4±0.5	88.5±0.8	88.8±0.6	0.081
		Women	93.7±0.3	93.8±0.3	93.3±0.5	91.6±1.0	91.8±0.6	0.028

Covariates: age, smoking, exercise, and waist circumference

**Table 3:** Logistic regression analysis for the lowest quintile of predicted FVC% and the lowest quintile of predicted FEV1% by fasting plasma glucose.

	Lowest quintile of predicted FVC% (<83%)				Lowest quintile of predicted FEV1% (<82%)			
	Model 1		Model 2		Model 1		Model 2	
Fasting plasma glucose (mg/dl)	Odds ratio (95% CI)	P	Odds ratio (95% CI)	P	Odds ratio (95% CI)	P	Odds ratio (95% CI)	P
-89	reference		reference		reference		reference	
90-99	1.05 (0.91-1.20)	0.505	1.01 (0.87-1.16)	0.948	0.97 (0.85-1.10)	0.622	0.92 (0.80-1.05)	0.222
100-109	1.08 (0.92-1.28)	0.346	1.12 (0.94-1.34)	0.198	0.97 (0.82-1.14)	0.679	0.97 (0.81-1.16)	0.728
110-125	1.50 (1.18-1.89)	0.001	1.34 (1.04-1.73)	0.026	1.34 (1.06-1.69)	0.015	1.22 (0.94-1.58)	0.138
diabetes	1.56 (1.30-1.88)	<0.001	1.61 (1.32-1.96)	<0.001	1.23 (1.02-1.48)	0.027	1.23 (1.01-1.52)	0.043

Model 1: adjusted for age, sex, smoking, exercise, and waist circumference

Model 2: model 1 after exclusion for individuals with diagnosed history of chronic obstructive pulmonary disease, or bronchial asthma, or coronary heart disease, or cerebrovascular accident, or cancer

## Results

A total of 9,223 participants were included in this study. These comprised 4,119 men and 5,104 women with the following characteristics; average age: 50 (19-90) years; BMI: 24.2±0.1 kg/m^2^; 20.5% were ex-smokers and 23.2% were current smokers; 10.1% had diabetes and 20.6% exhibited IFG; predicted FVC was 92.5±0.1%; and predicted FEV1 was 91.9±0.1%.

Clinical characteristics of the study participants were analyzed by sex (Table 1). Women were older than men (mean ± SEM, 50.7±0.2 vs. 49.2±0.2 years, P<0.001) Frequencies of heavy alcohol drinking, regular exercise, and diabetes, body mass index, waist circumference, systolic and diastolic blood pressures, and fasting plasma glucose values were higher in men than in women (P<0.001 for all of these variables). About 44% of men were current smokers, compared with about 7% of women (P<0.001). Predicted FVC% and predicted FEV1% were lower in men than in women (predicted FVC%, 91.6±0.2 vs. 93.3±0.2, P<0.001; predicted FEV1%, 90.0±0.2 vs. 93.4±0.2, P<0.001).

This study included comparisons of predicted FVC% by fasting plasma glucose level in each sex before and after adjustment for age, exercise, smoking, and waist circumference (Table 2, Figure 1). In both sexes, predicted FVC% decreased with increasing fasting plasma glucose (P for trend = 0.004 in men and <0.001 in women) after adjustment for the abovementioned variables (Table 2). Within a FPG range of 110-125 mg/dl (IFG2), predicted FVC% started to decrease in each sex (92.0±0.4% in IFG1 vs. 90.2±0.7% in IFG2, P = 0.032 in men; 93.1±0.5% in IFG1 vs. 91.1±0.9% in IFG2, P = 0.039 in women) (Figure 1).

In terms of associations with FPG level, predicted FEV1% showed a less consistent association with FPG level, as compared to predicted FVC%. In men, predicted FEV1% decreased with increasing fasting plasma glucose (P for trend <0.001) without adjustment. However, there was no decrease in predicted FEV1% with increasing fasting plasma glucose level (P for trend = 0.081) after adjustment for the abovementioned variables, although predicted FEV1% in IFG2 was significantly lower than that in IFG1 (90.4±0.5% in IFG1 vs. 88.5±0.8% in IFG2, P = 0.048). In women, there was no difference in predicted FEV1% according to fasting plasma glucose level (P for trend = 0.062) without adjustment. Although predicted FEV1% decreased with increasing fasting plasma glucose after adjustment for the abovementioned variables (P for trend = 0.028), there was no difference in predicted FEV1% between IFG1 and IFG2 (93.3±0.5% in IFG1 vs. 91.6±1.0% in IFG2, P = 0.116).

To examine the presence of an independent association with predicted FVC% or predicted FEV1%, logistic regression analyses for the lowest quintile of predicted FVC% (<83%) and the lowest quintile of predicted FEV1% (<82%) were conducted with age, sex, exercise, smoking, fasting plasma glucose level, and waist circumference as variables before (n = 9223) and after (n = 8324) exclusion of individuals with history of diagnosed chronic obstructive pulmonary disease (n = 70), bronchial asthma (n = 317), coronary heart disease (n = 189), cerebrovascular accident (n = 165), or cancer (n = 252) (Table 3). After adjustment for several clinical parameters, neither NFG2 nor IFG1 were associated with the lowest quintile of predicted FVC%. IFG2 and diabetes were independently associated with the lowest quintile of predicted FVC% (IFG2: odds ratio [95%CI], 1.50 [1.18-1.89], P = 0.001; diabetes: 1.56 [1.30-1.88], P<0.001) with NFG1 as a control. Even after exclusion of individuals with the abovementioned disease, IFG2 and diabetes were independently associated with the lowest quintile of predicted FVC% (IFG2: odds ratio [95%CI], 1.34 [1.04-1.73], P = 0.026; diabetes: 1.61 [1.32-1.96], P<0.001) with NFG1 as a control.

After adjustment for several clinical parameters, neither NFG2 nor IFG1 were associated with the lowest quintile of predicted FEV1%. IFG2 and diabetes were independently associated with the lowest quintile of predicted FEV1% (IFG2: odds ratio [95%CI], 1.34 [1.06-1.69], P = 0.015; diabetes: 1.23 [1.02-1.48], P = 0.027) with NFG1 as a control. After exclusion of individuals with the abovementioned disease, which may affect spirometry results, the significant association of IFG2 with the lowest quintile of FEV1% disappeared (odds ratio [95%CI], 1.22 [0.94-1.58], P = 0.138). However, even after these exclusions, diabetes was independently associated with the lowest quintile of predicted FEV1% (1.23 [1.01-1.52], P = 0.043) with NFG1 as a control.

In the analyses of diabetes patients (n = 934), after adjustment for age and sex, predictive FVC% by diabetes duration was 88.7±0.8% (mean ± SEM) in newly detected cases (n = 283), 88.0±0.7% in diabetes with a duration of ≤5 years (n = 313), 87.3±0.9% in diabetes with a duration of >5 and ≤10 years (n = 183), and 87.8±1.0% in diabetes with a duration of >10 years (n = 155). There was no association between diabetes duration and predictive FVC% in participants with diabetes (P trend = 0.692); however, predictive FVC% decreased with increased HbA1c (P trend = 0.017). After adjustment for age and sex, predictive FVC% values according to HbA1c in diabetes patients (n = 868) were 89.9±0.7% (mean ± SEM) for HbA1c ≤6.5% (n = 281), 86.9±0.6% for HbA1c of >6.5% and ≤8.0% (n = 400), 87.5±1.1% for HbA1c of >8.0% and ≤10% (n = 129), and 87.0±1.6% for HbA1c of >10% (n = 58). There was no difference in predictive FVC% among those with an HbA1c of >6.5% and ≤8.0%, HbA1c of >8.0% and ≤10% and HbA1c of >10% in *post hoc* comparisons. Predictive FVC% for HbA1c of >6.5% and ≤8.0% were values significantly lower than in HbA1c of <6.5% (P = 0.002). There was no difference in predictive FEV1% according to diabetes duration or HbA1c in participants with diabetes (data not shown).

## Discussion

The finding in this study of a decreased predicted FVC% in individuals with diabetes is compatible with several previous studies [3-7]. Moreover, after adjustment for several clinical parameters, the data illustrate that the predicted FVC% begins to decrease with increasing FPG in the higher IFG range. Even after exclusion of those with several clinical diseases that may affect the results of pulmonary function tests, the significance persisted. Therefore, the data suggest that predicted FVC% may begin to decrease in the higher IFG range (FPG of 110-125 mg/dl).

The current findings are consistent with previous studies of the association between diabetes and lung function in mainly Caucasian ethnic groups [4,5,18] and Japanese populations [19,20]. Moreover, a recent Korean study reported that restrictive pulmonary dysfunction is independently associated with type 2 diabetes [6]. In the present study, a decreased predicted FEV1% was associated with FPG level. However, this association was less consistent and depended on sex, the presence of covariates included in the analysis, and exclusion of patients with diseases that may affect pulmonary function test results, in contrast to the more consistent and stronger association of FPG with predicted FVC%. According to the results of several cross-sectional and longitudinal studies that reported an association of diabetes with lung function, the reduction in FVC is more consistent than that in FEV1 [3-7,21]. Similarly, several studies of the relationship between metabolic syndrome and lung function have reported that impaired pulmonary function of a particularly restrictive pattern was independently associated with metabolic syndrome [22-24]. Thyagarahan and associates [25] found that a larger decrement of FVC relative to FEV1 is associated with systemic inflammation. These studies may support our finding that FVC has a stronger association with glucose tolerance status than FEV1.

To our knowledge, few studies have addressed the relationship between lung function and IFG. The Framingham Heart Study and the third Korea National Health and Nutrition Examination Survey showed that a FPG >102 mg/dl is associated with pulmonary dysfunction [5,26]. However, in these studies, participants with abnormal fasting glucose (IFG and diabetes) were not subdivided by fasting glucose value, instead being classified as one group. In the Jackson Heart study, predicted FVC% was decreased in women with IGT, but not in men. After adjustment for waist circumference, this significance did not persist [12]. A Korean study of those who visited a health promotion center also did not find a relationship between restrictive lung disease and IFG using a multivariate analysis [6]. In the present study, participants were subdivided into five groups according to FPG level to determine the FPG cut-off value that affects predictive FVC%. The results demonstrate that predicted FVC% started to decrease in IFG2 (FPG range: 110-125 mg/dl) in a Korean adult population. We identified a ~2% difference in predictive FVC% between IFG1 and IFG2 subjects. It seems likely that such a small decrease has no significant effect on clinical outcome. However, in patients with pre-existing coronary heart disease or chronic lung disease this small change in FVC% could result in significant adverse clinical outcomes.

Much available data support an association between diabetes and pulmonary function, but the pathophysiological mechanism underlying this association is not clear. Several mechanisms, including chronic inflammation, microangiopathy, and autonomic neuropathy, have been suggested to explain the decline in pulmonary function associated with diabetes [7].

The reduced pulmonary function in diabetes has been suggested to be related to disease duration as well as blood glucose levels [4,27]. However, we did not find a significant association between diabetes duration and pulmonary function. Moreover, there was no strong association between pulmonary function and HbA1c. This finding may be explained, at least in part, by the characteristics of the diabetic population in this study, who exhibited relatively good glycemic control (HbA1c: 7.3±1.5%) and a relatively short duration of diabetes (5.5±6.8 years). However, the issue of statistical power cannot be ignored. With regard to the association between exposure to hyperglycemia and pulmonary dysfunction, it should be mentioned that HbA1C was analyzed only after it had been measured in the survey. Considering the limitation that previous glycemic control was not evaluated, it cannot be concluded that exposure to hyperglycemia in diabetes patients is not associated with pulmonary dysfunction. Recently the KORA F4 study showed a J-shaped association of clinical distal sensorimotor polyneuropathy with quartiles of 2-h post-challenge glucose, but not with fasting glucose and HbA1c levels [28]. Because post-challenge glucose values were not available in this study, we could not examine the relative importance of fasting vs. post prandial glucose for lung function. This will be an interesting issue for future study.

The main limitation of this study is that it was performed on a limited cross-section of the population. For clarification of a causal relationship between pulmonary function and pre-diabetes, a longitudinal prospective study is needed. In conclusion, we found that predicted FVC% might begin to decrease in pre-diabetes, particularly in the FPG range of 110-125 mg/dl, in a national representative sample of the Korean adult population. These results suggest that the association between pre-diabetes and pulmonary function needs to be considered in clinical practice. Moreover, larger prospective studies of the association between pulmonary dysfunction and pre-diabetes, as well as re-analysis of the datasets of previous cohort studies, are required.
